# Effect of standard and physiological cell culture temperatures on *in vitro* proliferation and differentiation of primary broiler chicken *pectoralis major* muscle satellite cells

**DOI:** 10.3389/fphys.2023.1288809

**Published:** 2023-11-16

**Authors:** Caroline R. Gregg, Brittany L. Hutson, Joshua J. Flees, Charles W. Starkey, Jessica D. Starkey

**Affiliations:** Department of Poultry Science, Auburn University, Auburn, AL, United States

**Keywords:** broiler chicken, muscle satellite cell, cell culture temperature, myogenic regulatory factor, apoptosis, myogenesis

## Abstract

Culture temperatures for broiler chicken cells are largely based on those optimized for mammalian species, although normal broiler body temperature is typically more than 3°C higher. The objective was to evaluate the effects of simulating broiler peripheral muscle temperature, 41°C, compared with standard temperature, 38°C, on the *in vitro* proliferation and differentiation of primary muscle-specific stem cells (satellite cells; SC) from the *pectoralis major* (PM) of broiler chickens. Primary SC cultures were isolated from the PM of 18-day-old Ross 708 × Yield Plus male broilers. SC were plated in triplicate, 1.8-cm^2^, gelatin-coated wells at 40,000 cells per well. Parallel plates were cultured at either 38°C or 41°C in separate incubators. At 48, 72, and 96 h post-plating, the culture wells were fixed and immunofluorescence-stained to determine the expression of the myogenic regulatory factors Pax7 and MyoD as well as evaluated for apoptosis using a TUNEL assay. After 168 h in culture, plates were immunofluorescence-stained to visualize myosin heavy chain and Pax7 expression and determine myotube characteristics and SC fusion. Population doubling times were not impacted by temperature (*p* ≥ 0.1148), but culturing broiler SC at 41°C for 96 h promoted a more rapid progression through myogenesis, while 38°C maintained primitive populations (*p* ≤ 0.0029). The proportion of apoptotic cells increased in primary SC cultured at 41°C (*p* ≤ 0.0273). Culturing at 41°C appeared to negatively impact fusion percentage (*p* < 0.0001) and tended to result in the formation of thinner myotubes (*p* = 0.061) without impacting the density of differentiated cells (*p* = 0.7551). These results indicate that culture temperature alters primary broiler PM SC myogenic kinetics and has important implications for future *in vitro* work as well as improving our understanding of how thermal manipulation can alter myogenesis patterns during broiler embryonic and post-hatch muscle growth.

## 1 Introduction

Hypertrophic growth of post-mitotic muscle fibers in young animals relies on populations of resident, muscle-specific stem cells, or satellite cells (SC; White et al., 2010). SC are capable of proliferating and fusing with existing muscle fibers to increase DNA content and allow for growth by expanding the potential for protein synthesis (Hall and Ralston, 1989). There is growing interest in evaluating SC isolated from the *pectoralis major* (PM) of modern broiler chickens *in vitro* to better understand broiler muscle growth, as well as develop practical methods to optimize meat yield. However, *in vitro* research on SC from current broiler strains is limited, and cell culture conditions are not well established in the existing literature. Many avian SC experimental protocols have been adapted from early work culturing murine SC (Bischoff, 1986), where the physiological body temperature of the donor animal is typically between 37°C and 38°C. Many researchers have adopted this range as a standard culture temperature for chicken SC (Matsuda et al., 1983; Halevy et al., 2004; Geiger et al., 2018; Tompkins et al., 2021). Yet, the average core body temperature of modern broilers is closer to 41°C (Giloh et al., 2012; Peebles et al., 2021), so this widely used standard cell culture temperature may not be reproducing physiological conditions of the broiler SC niche *in vivo*.

Increasing culture temperature above 38°C appears to increase both proliferation and differentiation of chicken SC (Harding et al., 2016). However, it is worth noting that the previously mentioned experiment was conducted with passaged SC isolated from broilers over 25 years ago (McFarland et al., 1997) that likely differ greatly from modern broiler strains, given the extensive genetic selection for muscle growth. Considering recent evidence that culture temperature impacts SC function in cells from fast-growing turkeys differently compared to cells from their heritage strain counterparts (Xu et al., 2022; Xu et al., 2023), evaluating SC from modern broilers may provide an updated understanding of the influence of temperature. In mammalian species, culturing SC continuously at temperatures above their respective physiological body temperature suppresses proliferation in primary swine cells (Park et al., 2023) and reduces myotube diameter in primary human cultures (Yamaguchi et al., 2010). Therefore, comparing the effects of a standard cell culture temperature versus a temperature that is closer to that of peripheral broiler muscle on SC proliferation and differentiation could help establish optimal culture conditions to properly evaluate modern broiler SC in future work. It was hypothesized that primary SC isolated from broiler chicken PM would have improved proliferation and greater SC fusion when cultured at 41°C.

## 2 Materials and methods

All procedures regarding live birds were approved by the Auburn University Institutional Animal Care and Use Committee (PRN 2020-3767).

### 2.1 Primary cell isolation

Primary muscle cell cultures were isolated from the pooled PM of 18-day-old Ross 708 × Yield Plus male broilers (*n* = 6 per replicate isolation). The broilers were reared on a common corn- and soybean meal-based diet formulated to meet or exceed primary breeder nutrient specifications in floor pens in an environmentally controlled facility (Charles C. Miller Jr. Poultry Research and Education Center, Auburn, AL, United States). Lighting and temperature were established according to primary breeder recommendations. Prior to muscle harvest and immediately following euthanasia via electrocution, a thermometer was inserted approximately 2–3 cm into a small opening cut in the center of the PM within a sterile environment. The average PM internal temperature of all birds harvested (*n* = 18) was 41.14 ± 0.08°C. PM was harvested, trimmed of excess fat, and briefly stored in Dulbecco’s modified Eagle’s medium (DMEM; Thermo Fisher, Waltham, MA, Cat. 10567022) with 1% antibiotic/antimycotic (AB/AM; Thermo Fisher, Waltham, MA, Cat. 15240112) + 0.1% gentamicin (Thermo Fisher, Waltham, MA, Cat. 15710064) at room temperature. PM tissue was pooled and chopped using a bullet chopper to a uniform consistency. SC isolation methods were adapted from Yablonka-Reuveni et al. (1987). Tissue was digested in 0.2% collagenase type 1 (Sigma-Aldrich, St. Louis, MO, Cat. C0130) in DMEM for 1 h with mechanical inversion every 5 min. Tissue was rinsed with phosphate-buffered saline (PBS) + 1% AB/AM + 0.1% gentamycin and then resuspended in PBS and vigorously shaken. The suspended tissue was centrifuged at 500 × g for 5 min, and then the cell-containing supernatant was collected and centrifuged at 1,500 × g for 5 min to obtain a cell pellet. This process was repeated three times to maximize cell yield, and the resulting cell pellets were resuspended in low-glucose DMEM (Thermo Fisher, Waltham, MA, Cat. 10567014) with 5% horse serum (Millipore Sigma, Burlington, MA, Cat. H1270) + 3% AB/AM + 0.3% gentamicin, pooled, and then passed through a 40-μM filter (Sigma-Aldrich, St. Louis, MO, Cat. SCNY00040). A density centrifugation step to remove muscle debris was conducted by layering the cell suspension over 20% Percoll (Cytiva, Marlborough, MA, Cat. 17089101) in Minimum Essential Medium (MEM; Thermo Fisher, Waltham, MA, Cat. 11090081) and centrifuged at 1,700 × g for 5 min in 15-mL conical tubes that were previously coated with horse serum. The remaining muscle debris layer was removed from the top of the tube and discarded, and cells were collected from the lower fraction. Cells were rinsed in MEM and resuspended in DMEM with 10% horse serum + 10% DMSO (Millipore Sigma, Burlington, MA, Cat. D2650) + 3% AB/AM + 0.3% gentamicin for long-term cryopreservation in liquid nitrogen until further analysis. Three replicate isolations were conducted to obtain three independent pools of cells from different birds of the same flock on the same day.

### 2.2 Cell culture

Upon removal from liquid nitrogen storage, vials of primary SC were briefly thawed in a water bath and rinsed in low-glucose DMEM. Live cells were quantified using the Countess™ 3 Automated Cell Counter (Invitrogen, Waltham, MA) via trypan blue dye exclusion staining (Invitrogen, Waltham, MA, Cat. T10282). Cells were diluted in proliferation media, as described in Flees et al. (2022), consisting of low-glucose DMEM with 10% chicken serum (Millipore Sigma, Burlington, MA, Cat. C5405) + 5% horse serum + 1% antibiotic/antimycotic + 0.1% gentamicin to achieve 40,000 live cells per well in triplicate on 24-well, gelatin-coated, tissue culture plates. The plates were incubated in one of two tri-gas Heracell™ 160i incubators (Thermo Fisher, Waltham, MA, Cat. 51033557) with 18% oxygen and 5% carbon dioxide set at either 38 or 41°C. For the proliferation and TUNEL assays, parallel plates were removed after 48, 72, and 96 h in culture. The final plates used for the differentiation assay were changed to differentiation media at 96 h post-plating consisting of low-glucose DMEM + 5% horse serum + 1% antibiotic/antimycotic + 0.1% gentamicin. The final parallel plates were removed after an additional 72 h or 168 h post-plating. The media were refreshed every 48 h. The experiment was replicated three times with replicate pools of cells from different birds of the same flock isolated on the same day.

### 2.3 Proliferation

The expression of myogenic regulatory factor (MRF) at 48, 72, and 96 h post-plating was determined using indirect immunofluorescence staining adapted from Day et al. (2007). In brief, cultures were rinsed with PBS before fixation in 4% paraformaldehyde (Santa Cruz Biotechnology, Dallas, TX, Cat. Sc-281692) for 10 min, followed by 15 min of permeabilization with 0.2% Triton X-100 (Thermo Fisher, Waltham, MA, Cat. A16046). Nonspecific binding was blocked using 3% bovine serum albumin (MP Biomedicals, Irvine, CA, Cat. 08810032) in PBS. Primary antibodies against the MRF paired box 7 (Pax7; Developmental Studies Hybridoma Bank, Iowa City, IA) and myogenic determination factor 1 (MyoD; Santa Cruz Biotechnology, Dallas, TX, Cat. sc-377460) were applied, followed by goat anti-mouse IgG1 Alexa Fluor 488 and IgG2b Alexa Fluor 546 (Invitrogen, Waltham, MA, Cat. A-21121 and A-21143). A 4′,6-diamidino-2-phenylindole (DAPI; MP Biomedicals, Santa Ana, CA, Cat. 0215757410) nuclear counterstain was applied to label all cell nuclei. Five random fields per culture well were captured using a Nikon ECLIPSE^®^ Ti2 inverted fluorescence microscopy system at ×200 magnification. The Taxonomy tool in NIS-Elements software was used to enumerate populations of Pax7+:MyoD-, Pax7+:MyoD+, Pax7+:MyoD+, and MRF+ (Pax7+:MyoD-, Pax7+:MyoD+, and Pax7+:MyoD+) nuclei. Populations were set as a proportion of total nuclei as well as reported on a mm^2^ basis. Population data were used to calculate doubling time using the following formula adapted from Li et al. (2011), where *t* = time in h, Nt = final cell count, and Ni = initial cell count.

$$DT=\frac{t}{\log_{2}\left(\frac{Nt}{Ni}\right)}.$$

### 2.4 Apoptosis

The incidence of late-stage apoptotic cells was determined at 48, 72, and 96 h post-plating on parallel plates using a Click-iT™ TUNEL Alexa Fluor Imaging Assay (Invitrogen, Waltham, MA, Cat. C10245) to identify DNA fragmentation associated with cell death. Extra culture wells were plated to serve as a positive control with DNase I treatment as per the manufacturer’s recommendations. A DAPI nuclear counterstain was applied to label all cell nuclei. Following completion of the assay protocol, five random fields per culture well were captured using a Nikon ECLIPSE^®^ Ti2 inverted fluorescence microscopy system at ×200 with the Taxonomy tool in NIS-Elements software to determine cells that were TUNEL-positive per mm^2^ as well as on a proportion of total nuclei.

### 2.5 Differentiation

At 168 h post-plating, indirect immunofluorescence staining was used to evaluate myogenic cell differentiation and fusion. The same protocol as the proliferation assay was employed using primary antibodies against Pax7 and myosin heavy chain (MHC; Developmental Studies Hybridoma Bank, Iowa City, IA) followed by goat anti-mouse IgG1 Alexa Fluor 488 and IgG2b Alexa Fluor 546 with a DAPI nuclear counterstain. Five representative fields per well were captured using a Nikon ECLIPSE^®^ Ti2 inverted fluorescence microscopy system at ×200 magnification. NIS-Elements software was used to analyze all images. Myotube boundaries were determined by MHC expression, and myotube width was measured in µm perpendicular to the widest point using the Length 3D tool in NIS-Elements. When myotubes branched off one another, each branch was considered a new myotube. The number of width measurements obtained per image was used to quantify the density of myotubes per mm^2^ as well as the coefficient of variation among the widths. The myotube area was quantified as a proportion of MHC expression to the total image area using the Binary feature of NIS-Elements software as previously described by Murphy et al. (2011) and Ferreira et al. (2020), with thresholds set for each image. Finally, SC fusion was determined using the Taxonomy tool in NIS-Elements to classify nuclei as either internal or external to the myotubes and calculated as a proportion of internal to total nuclei. The density of cells expressing Pax7 was also determined.

### 2.6 Statistical analysis

MRF heterogeneity and apoptosis data during proliferation were analyzed by time point, and doubling time, myotube characteristics, and fusion data were analyzed as a one-way analysis of variance using the generalized linear mixed model GLIMMIX procedure in SAS version 9.4 (PC version 9.4, SAS Inst. Inc., Cary, NC, United States). Culture temperature was the main effect. Culture well (*n* = 9 per treatment, assay, and time point) served as the experimental unit. The PDIFF option in SAS was used to perform all possible pairwise least square mean comparisons at *p* < 0.05. The Satterthwaite adjustment was used to correct the degrees of freedom. Proportional data were analyzed using the events/experiments syntax with a binomial distribution, and both continuous and proportional data were analyzed using an R-side covariance structure. Significant differences were declared when *p* ≤ 0.05 and tendencies when 0.0501 ≤ *p* ≤ 0.10.

## 3 Results

### 3.1 Proliferation

**FIGURE 1:**
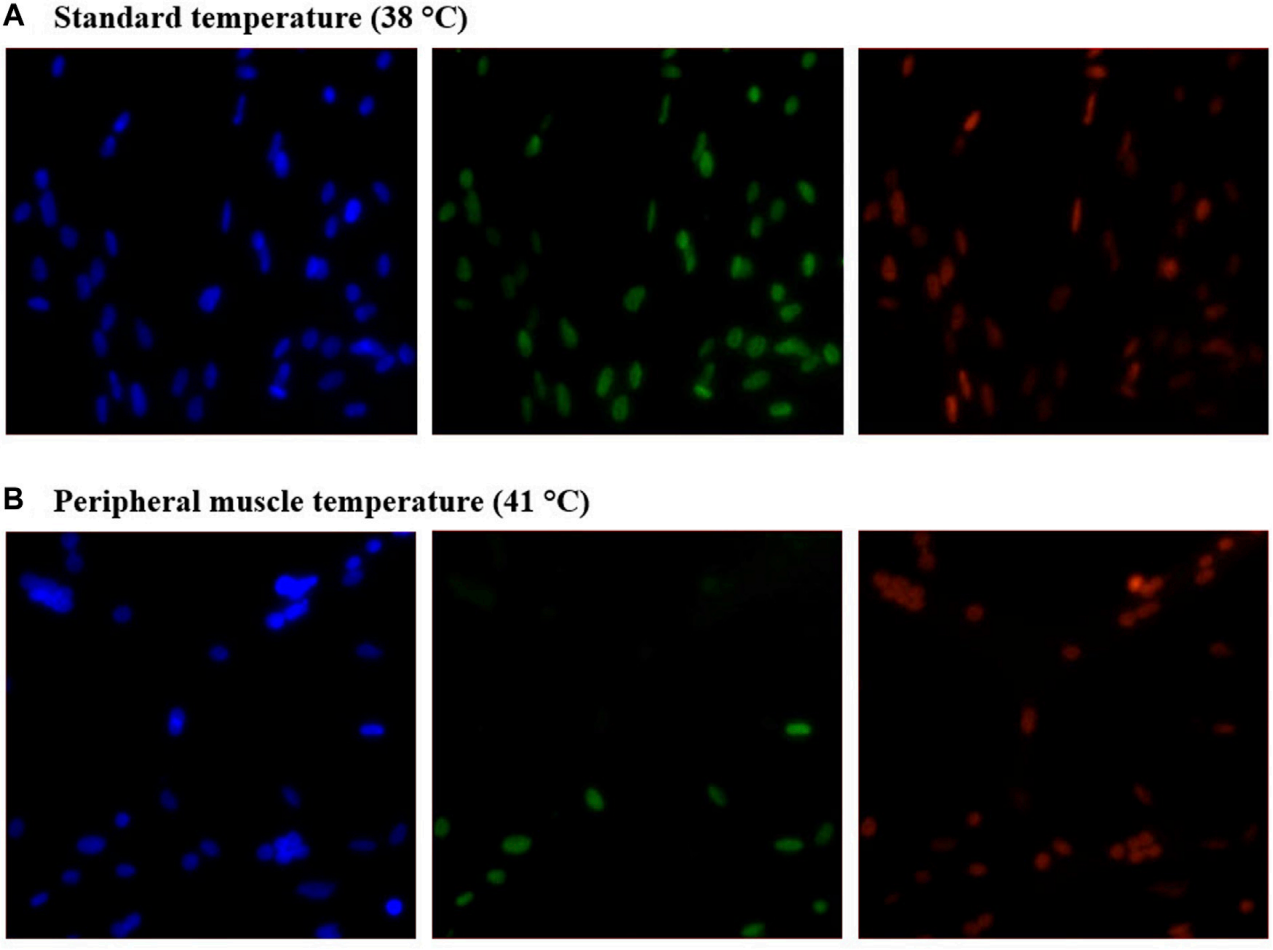
Effect of culture temperature on myogenic regulatory factor expression in primary broiler chicken *pectoralis major* satellite cell cultures after 96 h. Representative inverted fluorescence images of 4′,6-diamidino-2-phenylindole-stained nuclei (blue; left) and Pax7 (green; center) and MyoD (red; right) protein expression in cultures grown at 38°C **(A)** or 41°C **(B)**.

**FIGURE 2:**
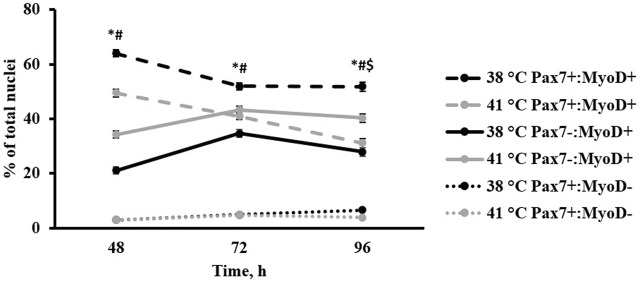
Effect of culture temperature on primary broiler chicken *pectoralis major* satellite cell myogenic regulatory factor heterogeneity over time. *n* = 9 wells per treatment of either 38 or 41°C incubation were immunofluorescence-stained to detect Pax7 and MyoD expression at 48, 72, and 96 h post-plating. Populations are presented as a percentage of total nuclei. Total nuclei describes every nucleus expressing the 4′,6-diamidino-2-phenylindole counterstain regardless of the status of other target proteins. Error bars represent the highest standard error of the pairwise mean comparison. Least square means accompanied by a * signify that the proportion of Pax7+:MyoD+ cells differs (*p* < 0.05) between 38°C and 41°C, # signify that the proportion of Pax7-:MyoD+ cells differs (*p* < 0.05) between 38°C and 41°C, and $ signify that the proportion of Pax7+:MyoD- cells differs (*p* < 0.05) between 38°C and 41°C.

Heterogeneity of Pax7+:MyoD-, Pax7-:MyoD+, and Pax7+:MyoD+ SC populations after 48, 72, and 96 h in culture are reported in Table 1. The density of total nuclei in the cultures was similar among temperatures at every time point (*p* ≥ 0.2041; Table 1). After 48 h in culture, 41°C conditions increased the density of Pax7-:MyoD+ (*p* = 0.0078; Table 1) and reduced the density of Pax7+:MyoD+ populations per mm^2^ (*p* = 0.0006; Table 1), which was reflected in the proportional data. After 72 h, only the density of the Pax7-:MyoD+ population was increased in wells cultured at 41°C per mm^2^ (*p* = 0.0204; Table 1), and the relative densities of Pax7-:MyoD+, Pax7+:MyoD+, and MRF + populations were altered by temperature (*p* ≤ 0.0304; Table 1). The heterogeneity of proliferative SC populations was the most impacted by temperature after 96 h, where increasing to physiological temperature reduced the density of Pax7+:MyoD- and Pax7+:MyoD+ cells (*p* ≤ 0.0016; Table 1) and increased the density of Pax7-:MyoD+ cells by approximately 150% (*p* < 0.0001; Table 1). Diminished Pax7+ expression at 41°C after 96 h is shown in Figure 1. At 96 h, the relative densities as a proportion of the total nuclei of all populations quantified were altered by temperature (*p* < 0.0001; Table 1). The MRF heterogeneity of SC populations was altered over time in culture (Figure 2). Across all time points, the density of MRF+ nuclei as well as total nuclei was similar across treatments (*p* > 0.5292; Table 1). Finally, the doubling time of all populations measured was not impacted by culture temperature (*p* > 0.1148; Table 2).

**TABLE 1:** Effect of culture temperature on myogenic regulatory factor heterogeneity during proliferation of primary broiler chicken *pectoralis major* satellite cells.

Cell population^1^	Time post-plating, h											
	48				72				96			
	Temperature, °C		SEM^2^	*p*-value	Temperature, °C		SEM	*p*-value	Temperature, °C		SEM	*p*-value
	38	41			38	41			38	41		
Density, cells per mm^2^												
Pax7+:MvoD-	4	3	0	0.4818	11	12	1	0.3063	23^a^	14^b^	2	0.0008
Pax7-:MyoD+	25^b^	37^a^	3	0.0078	77^b^	114^a^	11	0.0204	99^b^	152^a^	12	0.0029
Pax7+:MyoD+	77^a^	53^b^	5	0.0006	115	108	11	0.6747	184^a^	117^b^	15	0.0016
MRF+	106	93	7	0.2119	202	235	22	0.3117	307	284	26	0.5292
Total nuclei	121	108	8	0.2602	221	264	24	0.2041	356	378	30	0.6003
Relative density, % of total nuclei												
Pax7+:MvoD-	2.99	2.95	0.35	0.9387	4.86	4.71	0.37	0.7533	6.60^a^	3.81^b^	0.27	<0.0001
Pax7-:MyoD+	21.04^b^	34.25^a^	1.35	<0.000	34.66^b^	43.23^a^	1.40	<0.0001	27.89^b^	40.24^a^	1.52	<0.0001
Pax7+:MyoD+	64.03^1^	49.47^b^	1.40	<0.000	51.89^a^	40.96^b^	1.32	<0.0001	51.83^a^	31.08^b^	1.68	<0.0001
MRF+	87.96	86.57	0.92	0.2656	91.47^a^	88.88^b^	0.83	0.0304	86.3^a^	75.12^b^	1.68	<0.0001

1 Total nuclei describes every nuclei expressing the 4′,6-diamidino-2-phenylindole counterstain regardless of the status of other target proteins; MRF+ describes a combination of cells positive for Pax7, MyoD, or both Pax7 and MyoD.

2 SEM = highest standard error of the pairwise mean comparisons.

^a,b^Means within the same row with different superscripts differ *p* ≤ 0.05.

**TABLE 2:** Effect of culture temperature on population doubling time from 48 to 96 h post-plating of primary broiler chicken *pectoralis major* satellite cells.

Cell population	Temperature, °C		SEM^1^	*p*-value
	38	41		
Population doubling time, h				
Pax7+:MyoD-	24.28	31.08	31.08	0.5284
Pax7-:MyoD+	29.97	23.69	4.58	0.3466
Pax7+:MyoD+	45.16	58.46	7.30	0.2156
MRF+	34.00	31.98	2.25	0.5347
Total nuclei	32.17	28.09	1.73	0.1148

1 SEM = highest standard error of the pairwise mean comparisons.

### 3.2 Apoptosis

**FIGURE 3:**
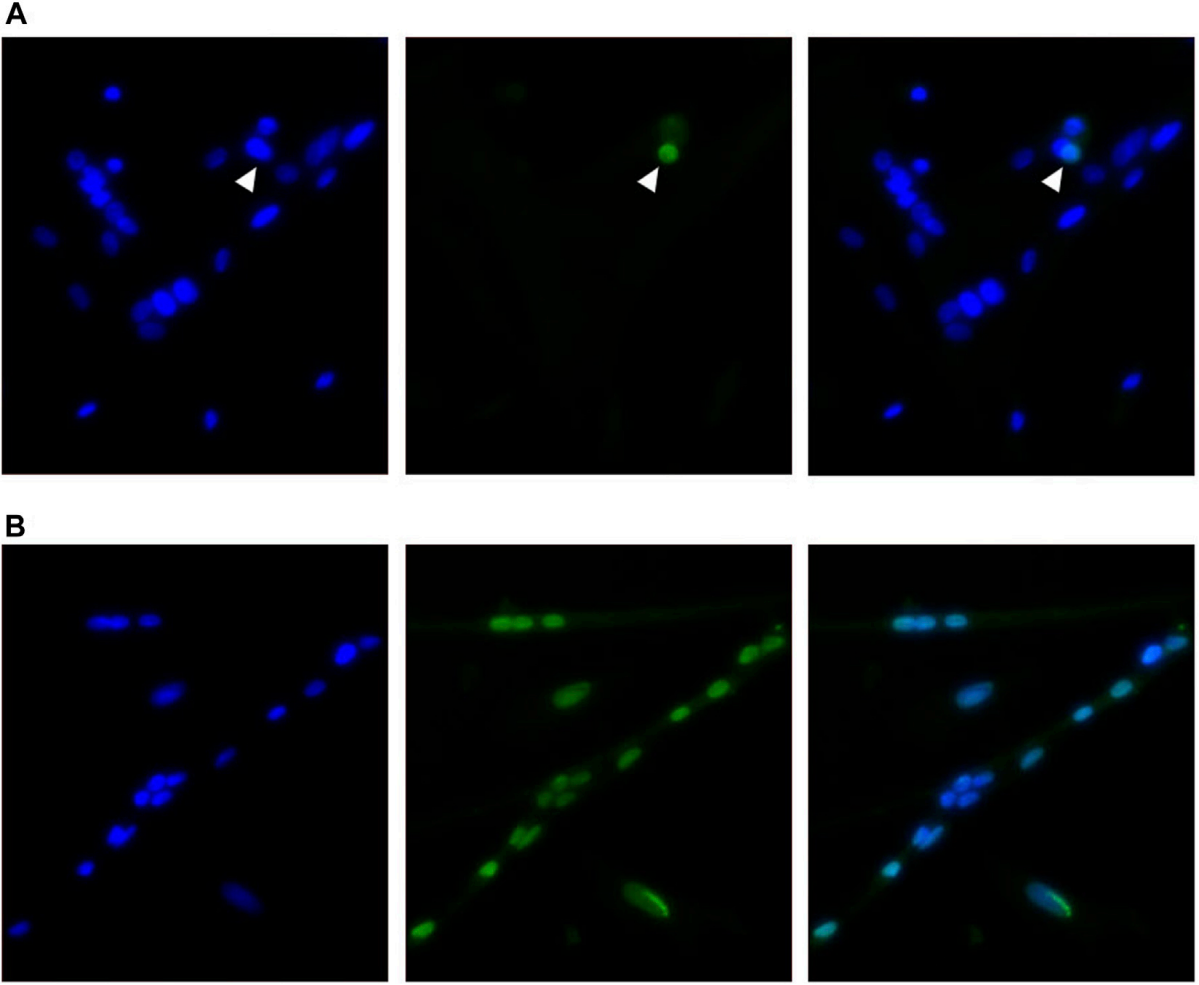
Representative inverted fluorescence images showing a TUNEL-positive nuclei indicated with an arrow among normal nuclei **(A)** in primary broiler *pectoralis major* satellite cell cultures with a corresponding positive control image **(B)** after DNase I treatment. Images show 4′,6-diamidino-2-phenylindole-stained nuclei (blue; left), TUNEL assay (green; center), and merged channels (right).

Cell apoptosis was measured during proliferation time points (48, 72, and 96 h post-plating) using a TUNEL assay to detect the presence of double-stranded DNA breaks that are characteristic of cells undergoing apoptosis. The results of this assay are shown in Table 3. Representative images showing a TUNEL-positive cell accompanied by images of positive control cells treated with DNase I are shown in Figure 3. The number of apoptotic cells per mm^2^ increased when cultured at 41°C after 48 and 96 h in culture compared with 38°C (*p* ≤ 0.0491; Table 3). In addition, 41°C incubation consistently increased the relative density of apoptotic cells (*p* ≤ 0.0302; Table 3) and diminished the relative density of normal cells (*p* ≤ 0.0273; Table 3) as a proportion of total nuclei at 48, 72, and 96 h post-plating. Regardless of treatment, apoptosis numerically reduced over time in culture from 48 to 96 h.

**TABLE 3:** Effect of culture temperature on apoptosis during proliferation of primary broiler chicken *pectoralis major* satellite cells.

Cell population^1^	Time post-plating, h											
	48				72				96			
	Temperature, °C		SEM^2^	*p*-value	Temperature, °C		SEM	*p*-value	Temperature, °C		SEM	*p*-value
	38	41			38	41			38	41		
Density, cells per mm^2^												
Normal	97	92	8	0.6431	248	226	18	0.3804	368	342	29	0.5401
Apoptotic	7^b^	10^a^	1	0.0491	11	13	1	0.1713	10^b^	13^a^	1	0.0255
Relative density, % of total nuclei												
Normal	93.23^a^	90.60^b^	0.69	0.0045	95.92^a^	94.64^b^	0.45	0.0302	97.45^a^	96.27^b^	0.30	0.0030
Apoptotic	6.79^a^	9.36^b^	0.68	0.0051	4.08^b^	5.39^a^	0.45	0.0273	2.54^b^	3.76	0.31	0.0026

1 Total nuclei describes every nuclei expressing the 4′,6-diamidino-2-phenylindole counterstain regardless of the cell status; normal describes nuclei not indicating double-stranded DNA breaks; apoptotic describes nuclei that were fluorescent under the TUNEL assay to denote apoptosis.

2 SEM = highest standard error of the pairwise mean comparisons.

^a,b^Means within the same row with different superscripts differ *p* ≤ 0.05.

### 3.3 Differentiation

**FIGURE 4:**
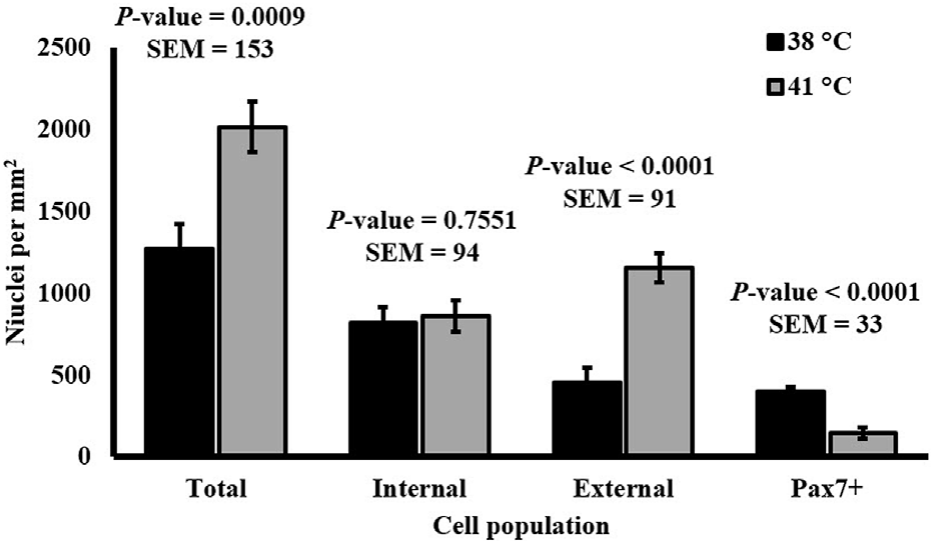
Effect of culture temperature on the density of nuclei relative to myotubes in primary broiler chicken *pectoralis major* satellite cell cultures after 168 h. *n* = 9 wells per treatment of either 38°C or 41°C incubation were immunofluorescence-stained to detect Pax7 and myosin heavy chain (MHC) expression. Total nuclei describes every nucleus expressing the 4′,6-diamidino-2-phenylindole counterstain regardless of the status of other target proteins; internal nuclei were located within an area expressing MHC protein; external nuclei did not overlap with MHC expression. Error bars represent the highest standard error of the pairwise mean comparison. ^a,b^Bars with different superscripts within a cell population differ *p* ≤ 0.05.

**FIGURE 5:**
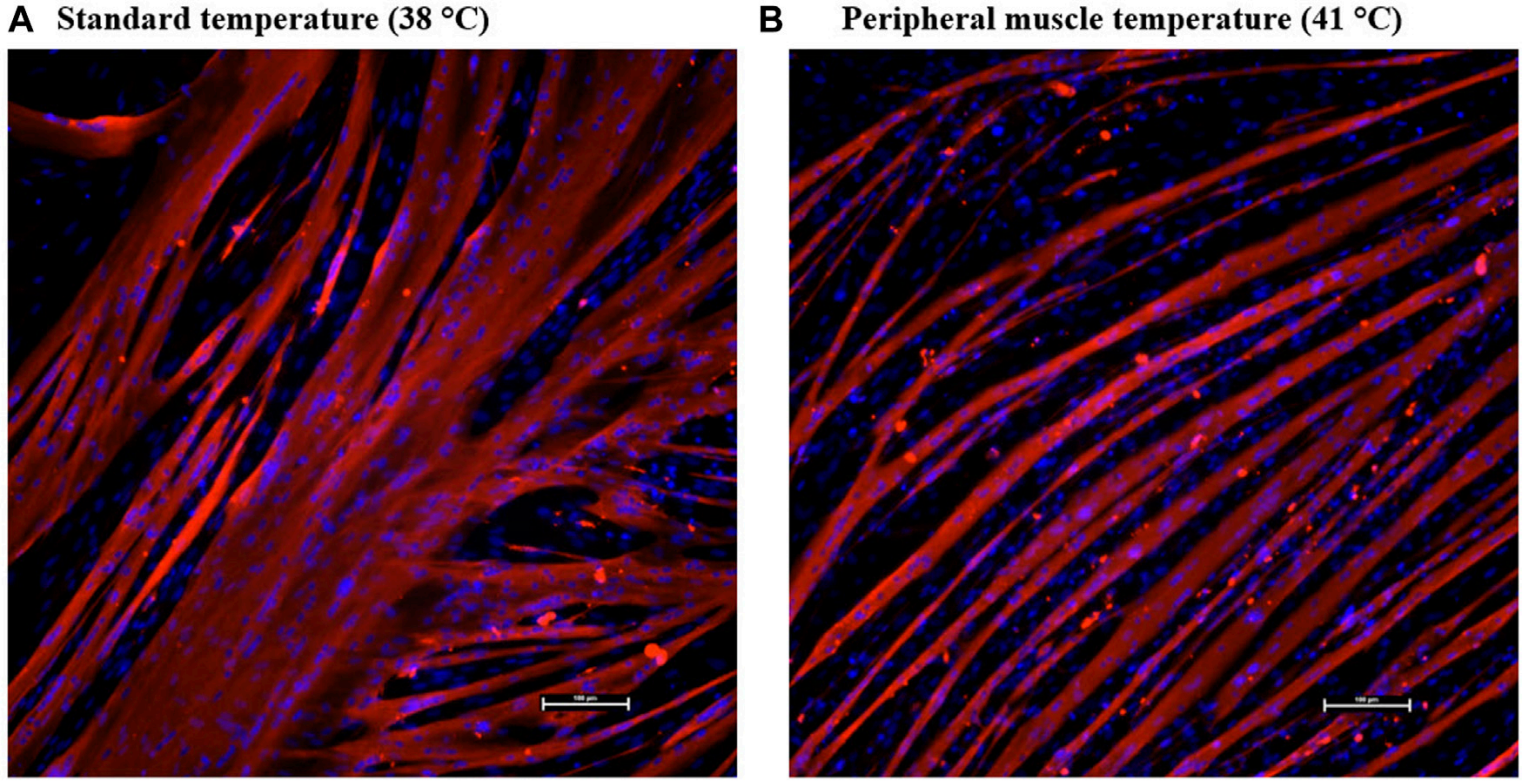
Effect of culture temperature on myotube formation in primary broiler chicken *pectoralis major* satellite cell cultures after 168 h. Representative inverted fluorescence images at ×200 magnification of myosin heavy chain-defined myotubes (red) and 4′,6-diamidino-2-phenylindole-stained nuclei (blue) in cultures grown at 38°C **(A)** or 41°C **(B)**. The scale bar depicts 100 µm.

SC differentiation was measured by visualizing MHC expression to determine the formation of myotubes in culture. DAPI+ nuclei that were inside the MHC boundaries were considered internal or fused cells, and those that were not overlapping with MHC were considered external or unfused cells. The expression of Pax7 was also determined. The percentage of fused myonuclei was greater in primary cultures grown at 38°C (*p* < 0.0001; Table 4). Interestingly, when internal nuclei were expressed on a mm^2^ basis, the density of fused nuclei was similar among treatments (*p* = 0.7551; Figure 4). The difference in fusion percentage appears to originate from a greater density of external nuclei at 41°C (*p* < 0.0001; Table 4), which was more than double the external nuclei present in cultures at 38°C. The density and proportion of Pax7+ SC populations were increased at 38°C (*p* < 0.0001; Table 4). The width of all myotubes was measured at the widest section of each myotube, and every new branch was considered its own myotube. Representative IF images of myotubes can be visualized in Figure 5. While the number of myotubes per mm^2^ was similar (*p* = 0.6111; Table 4), 41°C conditions tended to result in thinner myotubes (*p* = 0.0736; Table 4) that covered less total area of the culture well (*p* = 0.0806; Table 4). Myotubes appeared to be more consistent in width when cultured at 41°C and had a lower standard deviation (*p* = 0.0174; Table 4), but differences in the coefficient of variation among widths were not detected (*p* = 0.5248; Table 4).

**TABLE 4:** Effect of culture temperature on myonuclear fusion and myotube characteristics of primary broiler chicken *pectoralis major* satellite cells after 168 h in culture.

Variable^1^	Temperature, °C		SEM^2^	*p*-value
	38	41		
Nuclei per mm^2^	1272^b^	2017^a^	153	0.0009
Internal per mm^2^	819	860	94	0.7551
External per mm^2^	453^b^	1156^a^	91	<0.0001
Pax7+ per mm^2^	398^a^	144^b^	33	<0.0001
Pax7+, % of total nuclei	31.32^a^	7.15^b^	1.89	<0.0001
Fusion, % of total nuclei	64.37^a^	42.66^b^	2.72	<0.0001
Number of myotubes per mm^2^	64	67	3	0.6111
Average myotube width, µm	32^x^	24^y^	3	0.0736
Myotube width standard deviation, µm	32^a^	18^b^	4	0.0174
Myotube width coefficient of variation, %	75.09	72.27	3.31	0.5248
Myotube area, %	41.59^x^	33.58^y^	3.22	0.0806

1 Total nuclei describes every nucleus expressing the 4′,6-diamidino-2-phenylindole counterstain regardless of the status of other target proteins; internal nuclei were located within an area expressing the myosin heavy chain (MHC) protein; external nuclei did not overlap with MHC expression; myotube width was measured at the widest section of every new myotube branch as denoted by MHC expression; myotube area denotes the proportion of the culture well expressing MHC.

2 SEM = highest standard error of the pairwise mean comparisons.

^a,b^Means within the same row with different superscripts differ *p* ≤ 0.05.

^x,y^Means within the same row with different superscripts describe a tendency 0.10 > *p* < 0.05.

## 4 Discussion

Alterations to the MRF heterogeneity of SC populations at 48, 72, and 96 h post-plating indicate an overall shift in proliferative myogenic cell activity as culture temperature changes. While the MRF Pax7 and MyoD are both generally considered to be expressed during myoblast proliferation (Cornelison and Wold, 1997; Bentzinger et al., 2012), Pax7 expression is associated with primitive SC populations during early activation (Seale et al., 2000). MyoD expression is upregulated as the cell progresses toward commitment to the myogenic fate (Berkes and Tapscott, 2005) and appears to be required for differentiation, serving as the molecular switch from proliferation to differentiation (Cornelison et al., 2000). Based on the increased presence of Pax7+:MyoD- cells when culturing at 38°C during both proliferation and differentiation assays, this temperature may better retain a robust Pax7+:MyoD population or a population of SC either in the early stages of myogenesis or undergoing self-renewal. Based on the results of the differentiation assay, the observed increase in Pax7 expression at 38°C continued to 168 h post-plating. Culturing at 41°C advanced cells through the myogenic program faster as the presence of cells expressing MyoD was enhanced at that temperature (Figure 2). These data suggest that culturing primary broiler SC at physiological muscle temperature promoted a more rapid progression toward myogenic differentiation. However, in disagreement with the original hypothesis, total cell proliferation did not appear to be impacted by these culture temperatures as the densities of total nuclei and nuclei expressing one or more MRF remained similar over time. This was also reflected by similarities in the doubling time of all populations measured among temperatures between 48 and 96 h. It should be noted that proliferative capability was not altered during the proliferation time points between 48 and 96°h, but total nuclei was distinctly increased at 168 h post-plating at 41°C. This could mean proliferation was impacted by temperature but occurring later than expected, thus warranting further analysis in the future. Hayashi and Yonekura (2019) reported an increase in the differentiation of C2C12 mouse myoblasts with a 2°C increase in culture temperature above physiological temperature with thermal stimulation at later time points compared with higher temperature during the first few days of culture only. This suggests a temporal component to the impact of temperature on myogenic cells and may help explain the increase in nuclei in cultures that occurred between 96 and 168 h observed here.

Given that the peripheral muscle temperature of the PM harvested for the primary cultures utilized in this experiment was 41°C, it was hypothesized that culturing the cells at 41°C would better support proliferation and differentiation than the standard 38°C culture temperature since the increased temperature better replicates physiological conditions. This hypothesis was formulated based on observations by Harding et al. (2016) who reported increased DNA and creatine kinase content in broiler SC cultured at 43°C compared with a 38°C control temperature, indicating that increasing culture temperature improves cell proliferation and differentiation. These results were mirrored by a similar study with SC isolated from turkey PM that found that increasing culture temperature from 38°C to 41°C increased DNA content as well as resulted in the formation of wider myotubes (Clark et al., 2016). Furthermore, Xu et al. (2021) also reported increased proliferation and differentiation of turkey SC, but they also highlighted elevated gene expression of MyoD with increased culture temperature, which was consistent with observations from the present study. Researchers have previously attempted to relate the impact of elevated culture temperature on broiler SC to the impact of heat stress on muscle growth in the bird, yet the culture conditions they consider to be thermal stress may be better replicating the normal, physiological SC environment.

The results obtained from the present experiment appear to contradict the past literature as well as lead to a partial rejection of the original hypothesis as total cell proliferation was not impacted by temperature between the typical proliferation time points of 48 and 96 h post-plating, but MRF expression and fusion percentage were altered. One potential reason for the discrepancy between the results of this experiment and those reported previously may be the genetic difference between the cells from donor birds in the previous studies compared with cells from modern broilers presented here. Given that cells used for previously mentioned experiments originated from broilers and turkeys over two decades ago (McFarland et al., 1997; Velleman et al., 2000), it is possible that the rigorous selection pressure on increasing PM yield has altered the SC populations in modern broilers and turkeys since then. It has been demonstrated that the heat production of broilers has changed over time, as demonstrated by increasing heat production in modern *ad libitum*-fed broiler breeder hens compared with earlier genetic lines (Carney et al., 2022), and this may contribute to SC populations in modern broilers responding differently to temperature than their predecessor strains as the cellular environment and metabolism of the muscle evolve. The previous *in vitro* work was also conducted using SC after multiple passages, which would result in a highly selected population of SC that are less representative of the resident SC population *in vivo*. In addition, donor bird age may also impact how cells respond to temperature and aid in explaining the inconsistency in findings. Sarais et al. (2023) reported that the gene expression of SC from younger pigs with an undeveloped thermoregulation capacity responded to heat and cold stress *in vitro* differently than their older, developed counterparts. Therefore, the age of donor broilers and their capability to thermoregulate may also impact how SC function when cultured in different temperature environments.

Apoptosis was also evaluated at these time points and appeared to increase in cells cultured at a higher temperature. These results are in agreement with previous literature that found increased markers of apoptosis in cultured chicken SC at 41°C compared with 37°C (Siddiqui et al., 2020b) and at 43°C compared with 38°C (Harding et al., 2015). However, the proportion of apoptotic cells observed was overall higher than expected, which may be attributed to the use of freshly isolated (i.e., non-passaged) primary cultures that underwent minimal purification or selection apart from density gradient centrifugation. The TUNEL assay in the present study was conducted on parallel plates separate from MRF evaluation, so it was unfortunately impossible to determine whether apoptotic cells were also expressing myogenic markers and the exact cell populations more affected by apoptosis in the primary culture may be different than the proliferative SC populations of interest. The increase in apoptosis observed during proliferation time points was likely not detrimental for primary broiler SC at 41°C as total cells per mm^2^ were increased by 168 h post-plating in cultures grown at a higher temperature. Analysis of cell death during differentiation time points at these culture temperatures should be conducted to aid in understanding this observation.

Observing a similar density of fused cells across temperature treatments indicates a similar SC fusion capacity (Figure 4). The substantial difference in the densities of unfused cells raises questions about the identity of external cell populations. Culturing at 41°C may increase the proliferation of alternative cell populations without impacting the extent of SC fusion. It is impossible to determine whether all external cells were myogenic in nature as only the expression of Pax7 was assessed, and evaluating a more complete MRF panel would be needed. However, the relative density of cells expressing Pax7 was diminished at 41°C, indicating a reduction in primitive SC populations. It is possible that the unfused cell populations were myogenic in nature and capable of proliferation, but the increased temperature had prevented myotube fusion. It is also possible that these cells were non-myogenic populations like fibro/adipogenic progenitors, as primary muscle cultures likely contain non-myogenic progenitor populations. This is supported by a previous study comparing 37°C–41°C culture temperature on broiler fibroblasts that observed greater fibroblast proliferation at 41°C based on cell cycle analysis (Siddiqui et al., 2020a) and observations of improved chicken fibroblast *in vitro* adherence at 41°C (Kim et al., 2022). The external cells in the present study may have also been adipogenic in nature, as Harding et al. (2015) observed increased expression of adipogenic genes in broiler SC cultures grown at higher temperatures. This may be further supported by observations of diminished myofiber diameter and increased lipid content in cultured SC isolated from young broilers reared under continuous heat stress conditions compared with commercial conditions (Piestun et al., 2017). Further investigation is needed to characterize the identity and lineage of all progenitor cell types present in primary broiler PM cultures in response to altering temperatures.

Visually, myotubes cultured at physiological muscle temperature were formed and arranged differently than those at the standard lower temperature. The representative images in Figure 5 display a more regular alignment of thinner myotubes at 41°C, while those at the standard lower temperature appeared to be thicker and more variable. This was confirmed by quantifying myotube width and area. Myotubes at 41°C tended to be thinner and more consistent in shape and size than those cultured at 38°C. The lower culture temperature appeared to produce thicker myotubes that take up more total area of the culture well. The appearance of larger myotubes may explain the use of a temperature that is lower than the physiological culture temperature in previous literature, as thicker myotubes may be perceived as more desirable for *in vitro* experiments. With the retention of a robust Pax7+:MyoD- population and genesis of thicker myotubes promoting the fusion of a greater proportion of cells present, SC cultured at a lower temperature appeared to retain a more primitive status for longer, thus better replicating embryonic myogenesis (Sieiro-Mosti et al., 2014) despite being isolated from post-hatch broilers. The industry standard temperature for broiler egg incubation is typically 37.5°C. This has been shown to result in eggshell temperatures between 38°C and 39°C that were strongly correlated with embryo temperatures during later stages of incubation (Agyekum et al., 2022) and embryo temperatures between 37.5°C and 38°C during earlier stages (Tejeda et al., 2021). It has been previously established that the proliferation capacity of SC is greater in younger animals (Schultz and Lipton, 1982), and these results may reveal that placing SC from mature muscle into an embryonic-simulating environment enhanced the population of cells expressing transcription factors indicative of early proliferation. Moreover, these *in vitro* observations may serve to highlight and explain changes in broiler muscle growth following thermal manipulations during embryogenesis.

The results of embryonic thermal manipulation on post-hatch broiler performance are not consistent in the current literature. During the early stages of embryogenesis, increasing incubation temperature above the industry standard did not alter broiler body weight and breast yield in one study (Wall et al., 2022) but increased body weight and breast yield of similarly aged broilers in another (Janisch et al., 2015). While increasing temperature during incubation during late-stage development has been found to reduce broiler body weight (Piestun et al., 2008; Piestun et al., 2015), increased temperature on embryonic days 16–18 has also been observed to improve the reservoir of myogenic cells, increasing Pax7 expression and PM yield (Piestun et al., 2009). A high eggshell temperature during development has also been found to improve breast meat yield without impacting the overall growth performance (Molenaar et al., 2011). These data support the theory that pre-hatch thermal manipulation can alter muscle growth as increased temperature *in vitro* impacted broiler SC heterogeneity and myotube characteristics. The tendency for a reduction in myotube width with a similar density of fused nuclei when culturing at a higher temperature (Table 4; Figure 4) suggests that temperature may impact the density of myonuclei per fiber. When applied *in vivo*, these potential changes could lead to long-term alterations in mature muscle hypertrophy. Previously, thermal manipulation during critical points in myogenesis was found to increase the total muscle fiber number in turkeys (Maltby et al., 2004), and increasing temperature of cultured bovine SC altered myosin heavy chain isoform expression, implying that temperature may also alter the fiber type during development (Kim and Kim, 2023). Differences in myotube width observed in the present study may help understand how increased temperature during embryonic myogenesis changes SC differentiation patterns that could lead to increased fiber number. Further investigation to elucidate the mechanisms through which temperature impacts broiler PM SC function is warranted.

In conclusion, incubation of primary broiler chicken PM SC at 38°C and 41°C impacted MRF heterogeneity and myotube formation. Previous literature evaluating the impact of culturing broiler SC at temperatures above 38°C referred to elevated temperatures as thermal stress conditions and attempted to relate this to potential impacts of heat stress in post-hatch broiler muscle (Harding et al., 2015; Harding et al., 2016; Siddiqui et al., 2020b). However, a culture temperature closer to 41°C may better replicate the physiological environment of broiler SC, which stresses the importance of avoiding misrepresentations of *in vitro* experimental results when applying conclusions back to *in vivo* myogenesis. Furthermore, the observed shift in SC heterogeneity and changes in myotube characteristics may help understand the mechanism through which thermal manipulation during embryonic myogenesis can influence broiler muscle yield.

## Data Availability

The raw data supporting the conclusion of this article will be made available by the authors, without undue reservation.

## References

[B1] Agyekum G. Okai M. A. Tona J. K. Donkoh A. Hamidu J. A. (2022). Impact of incubation temperature profile on chick quality, bone, and immune system during the late period of incubation of Cobb 500 broiler strain. Poult. Sci. 101 (9), 101999. 10.1016/j.psj.2022.101999 35841642 PMC9289892

[B2] Bentzinger C. F. Wang Y. X. Rudnicki M. A. (2012). Building muscle: molecular regulation of myogenesis. Cold Spring Harb. Perspect. Biol. 4 (2), a008342. 10.1101/cshperspect.a008342 22300977 PMC3281568

[B3] Berkes C. A. Tapscott S. J. (2005). MyoD and the transcriptional control of myogenesis. Semin. Cell Dev. Biol. 16 (4-5), 585–595. 10.1016/j.semcdb.2005.07.006 16099183

[B4] Bischoff R. (1986). Proliferation of muscle satellite cells on intact myofibers in culture. Dev. Biol. 115 (1), 129–139. 10.1016/0012-1606(86)90234-4 3516758

[B5] Carney V. L. Anthony N. B. Robinson F. E. Reimer B. L. Korver D. R. Zuidhof M. J. (2022). Evolution of maternal feed restriction practices over 60 years of selection for broiler productivity. Poult. Sci. 101 (10), 101957. 10.1016/j.psj.2022.101957 35973347 PMC9395665

[B6] Clark D. L. Coy C. S. Strasburg G. M. Reed K. M. Velleman S. G. (2016). Temperature effect on proliferation and differentiation of satellite cells from turkeys with different growth rates. Poult. Sci. 95 (4), 934–947. 10.3382/ps/pev437 26769270

[B7] Cornelison D. Olwin B. B. Rudnicki M. A. Wold B. J. (2000). MyoD−/− satellite cells in single-fiber culture are differentiation defective and MRF4 deficient. Dev. Biol. 224 (2), 122–137. 10.1006/dbio.2000.9682 10926754

[B8] Cornelison D. Wold B. J. (1997). Single-cell analysis of regulatory gene expression in quiescent and activated mouse skeletal muscle satellite cells. Dev. Biol. 191 (2), 270–283. 10.1006/dbio.1997.8721 9398440

[B48] Day K. Shefer G. Richardson J. B. Enikolopov G. Yablonka-Reuveni Z. (2007). Nestin-GFP reporter expression defines the quiescent state of skeletal muscle satellite cells. cells. Dev. Biol. 304 (1), 246–259. 10.1016/j.ydbio.2006.12.026 17239845 PMC1888564

[B49] Flees J. J. Starkey C. W. Starkey J. D. (2022). Effect of different basal culture media and sera type combinations on primary broiler chicken muscle satellite cell heterogeneity during proliferation and differentiation. Animals (Basel) 12 (11). 10.3390/ani12111425 PMC9179426 35681889

[B9] Ferreira T. Z. Kindlein L. Flees J. J. Shortnacy L. K. Vieira S. L. Nascimento V. P. (2020). Characterization of Pectoralis major muscle satellite cell population heterogeneity, macrophage density, and collagen infiltration in broiler chickens affected by Wooden Breast. Front. Physiol. 11, 529. 10.3389/fphys.2020.00529 32536877 PMC7268892

[B10] Geiger A. E. Daughtry M. R. Gow C. M. Siegel P. B. Shi H. Gerrard D. E. (2018). Long-term selection of chickens for body weight alters muscle satellite cell behaviors. Poult. Sci. 97 (7), 2557–2567. 10.3382/ps/pey050 29617946

[B11] Giloh M. Shinder D. Yahav S. (2012). Skin surface temperature of broiler chickens is correlated to body core temperature and is indicative of their thermoregulatory status. Poult. Sci. 91 (1), 175–188. 10.3382/ps.2011-01497 22184442

[B12] Halevy O. Piestun Y. Allouh M. Z. Rosser B. W. Rinkevich Y. Reshef R. (2004). Pattern of Pax7 expression during myogenesis in the posthatch chicken establishes a model for satellite cell differentiation and renewal. Dev. Dyn. 231 (3), 489–502. 10.1002/dvdy.20151 15390217

[B13] Hall Z. W. Ralston E. (1989). Nuclear domains in muscle cells. Cell 59 (5), 771–772. 10.1016/0092-8674(89)90597-7 2686838

[B14] Harding R. L. Clark D. L. Halevy O. Coy C. S. Yahav S. Velleman S. G. (2015). The effect of temperature on apoptosis and adipogenesis on skeletal muscle satellite cells derived from different muscle types. Physiol. Rep. 3 (9), e12539. 10.14814/phy2.12539 26341996 PMC4600383

[B15] Harding R. L. Halevy O. Yahav S. Velleman S. G. (2016). The effect of temperature on proliferation and differentiation of chicken skeletal muscle satellite cells isolated from different muscle types. Physiol. Rep. 4 (8), e12770. 10.14814/phy2.12770 27125667 PMC4848725

[B16] Hayashi S. Yonekura S. (2019). Thermal stimulation at 39°C facilitates the fusion and elongation of C2C12 myoblasts. Anim. Sci. J. 90 (8), 1008–1017. 10.1111/asj.13227 31134721

[B17] Janisch S. Sharifi A. R. Wicke M. Krischek C. (2015). Changing the incubation temperature during embryonic myogenesis influences the weight performance and meat quality of male and female broilers. Poult. Sci. 94 (10), 2581–2588. 10.3382/ps/pev239 26316339

[B18] Kim S. H. Kim C. J. Lee E. Y. Son Y. M. Hwang Y. H. Joo S. T. (2022). Optimal pre-plating method of chicken satellite cells for cultured meat production. Food Sci. Anim. Resour. 42 (6), 942–952. 10.5851/kosfa.2022.e61 36415580 PMC9647181

[B19] Kim W. S. Kim J. (2023). Exploring the impact of temporal heat stress on skeletal muscle hypertrophy in bovine myocytes. J. Therm. Biol. 117, 103684. 10.1016/j.jtherbio.2023.103684 37625343

[B20] Li J. Gonzalez J. M. Walker D. K. Hersom M. J. Ealy A. D. Johnson S. E. (2011). Evidence of heterogeneity within bovine satellite cells isolated from young and adult animals. J. Anim. Sci. 89 (6), 1751–1757. 10.2527/jas.2010-3568 21297061

[B21] Maltby V. Somaiya A. French N. A. Stickland N. C. (2004). In ovo temperature manipulation influences post-hatch muscle growth in the Turkey. Br. Poult. Sci. 45 (4), 491–498. 10.1080/00071660412331286190 15484723

[B22] Matsuda R. Spector D. H. Strohman R. C. (1983). Regenerating adult chicken skeletal muscle and satellite cell cultures express embryonic patterns of myosin and tropomyosin isoforms. Dev. Biol. 100 (2), 478–488. 10.1016/0012-1606(83)90240-3 6653881

[B23] McFarland D. C. Gilkerson K. K. Pesall J. E. Ferrin N. H. Wellenreiter R. H. (1997). *In vitro* characteristics of myogenic satellite cells derived from the pectoralis major and biceps femoris muscles of the chicken. Cytobios 91 (364), 45–52. 9569620

[B24] Molenaar R. Hulet R. Meijerhof R. Maatjens C. M. Kemp B. van den Brand H. (2011). High eggshell temperatures during incubation decrease growth performance and increase the incidence of ascites in broiler chickens. Poult. Sci. 90 (3), 624–632. 10.3382/ps.2010-00970 21325234

[B25] Murphy M. M. Lawson J. A. Mathew S. J. Hutcheson D. A. Kardon G. (2011). Satellite cells, connective tissue fibroblasts and their interactions are crucial for muscle regeneration. Development 138 (17), 3625–3637. 10.1242/dev.064162 21828091 PMC3152921

[B26] Park J. Lee J. Shim K. (2023). Effects of heat stress exposure on porcine muscle satellite cells. J. Therm. Biol. 114, 103569. 10.1016/j.jtherbio.2023.103569 37344027

[B27] Peebles E. D. Oliveira T. F. B. Kim E. J. Olojede O. C. Elliott K. E. C. Lindsey L. L. (2021). Research Note: effects of the in ovo injection of organic zinc, manganese, and copper and posthatch holding time before placement on broiler body temperature during grow out. Poult. Sci. 100 (2), 755–759. 10.1016/j.psj.2020.10.048 33518129 PMC7858143

[B28] Piestun Y. Harel M. Barak M. Yahav S. Halevy O. (2009). Thermal manipulations in late-term chick embryos have immediate and longer term effects on myoblast proliferation and skeletal muscle hypertrophy. J. Appl. Physiol. (1985) 106(1), 233–240. 10.1152/japplphysiol.91090.2008 19023019 PMC2636946

[B29] Piestun Y. Patael T. Yahav S. Velleman S. G. Halevy O. (2017). Early posthatch thermal stress affects breast muscle development and satellite cell growth and characteristics in broilers. Poult. Sci. 96 (8), 2877–2888. 10.3382/ps/pex065 28444312

[B30] Piestun Y. Shinder D. Ruzal M. Halevy O. Brake J. Yahav S. (2008). Thermal manipulations during broiler embryogenesis: effect on the acquisition of thermotolerance. Poult. Sci. 87 (8), 1516–1525. 10.3382/ps.2008-00030 18648043

[B31] Piestun Y. Yahav S. Halevy O. (2015). Thermal manipulation during embryogenesis affects myoblast proliferation and skeletal muscle growth in meat-type chickens. Poult. Sci. 94 (10), 2528–2536. 10.3382/ps/pev245 26316337

[B32] Sarais F. Metzger K. Hadlich F. Kalbe C. Ponsuksili S. (2023). Transcriptomic response of differentiating porcine myotubes to thermal stress and donor piglet age. Int. J. Mol. Sci. 24 (17), 13599. 10.3390/ijms241713599 37686405 PMC10487455

[B33] Schultz E. Lipton B. H. (1982). Skeletal muscle satellite cells: changes in proliferation potential as a function of age. Mech. Ageing Dev. 20 (4), 377–383. 10.1016/0047-6374(82)90105-1 7166986

[B34] Seale P. Sabourin L. A. Girgis-Gabardo A. Mansouri A. Gruss P. Rudnicki M. A. (2000). Pax7 is required for the specification of myogenic satellite cells. Cell 102 (6), 777–786. 10.1016/s0092-8674(00)00066-0 11030621

[B35] Siddiqui S. H. Subramaniyan S. A. Kang D. Park J. Khan M. Choi H. W. (2020a). Direct exposure to mild heat stress stimulates cell viability and heat shock protein expression in primary cultured broiler fibroblasts. Cell Stress Chaperones 25 (6), 1033–1043. 10.1007/s12192-020-01140-x 32696180 PMC7591668

[B36] Siddiqui S. H. Subramaniyan S. A. Kang D. Park J. Khan M. Shim K. (2020b). Modulatory effect of heat stress on viability of primary cultured chicken satellite cells and expression of heat shock proteins *ex vivo* . Anim. Biotechnol. 32, 774–785. 10.1080/10495398.2020.1757460 32340526

[B37] Sieiro-Mosti D. De La Celle M. Pele M. Marcelle C. (2014). A dynamic analysis of muscle fusion in the chick embryo. Development 141 (18), 3605–3611. 10.1242/dev.114546 25183875

[B38] Tejeda O. J. Meloche K. J. Starkey J. D. (2021). Effect of incubator tray location on broiler chicken growth performance, carcass part yields, and the meat quality defects wooden breast and white striping. Poult. Sci. 100 (2), 654–662. 10.1016/j.psj.2020.10.035 33518119 PMC7858132

[B39] Tompkins Y. H. Su S. Velleman S. G. Kim W. K. (2021). Effects of 20(S)-hydroxycholesterol on satellite cell proliferation and differentiation of broilers. Poult. Sci. 100 (2), 474–481. 10.1016/j.psj.2020.10.032 33518099 PMC7858162

[B40] Velleman S. G. Liu X. Nestor K. E. McFarland D. C. (2000). Heterogeneity in growth and differentiation characteristics in male and female satellite cells isolated from Turkey lines with different growth rates. Comp. Biochem. Physiology Part A Mol. Integr. Physiology 125 (4), 503–509. 10.1016/s1095-6433(00)00178-1 10840226

[B41] Wall B. L. Rueda M. S. Davis J. D. Purswell J. L. Starkey C. W. Starkey J. D. (2022). “Evaluation of thermal variation during early-stage incubation on broiler chicken growth performance, carcass characteristics, and the breast meat quality defects, Wooden Breast and White Striping,” in Poultry science association annual meeting (Texas): San Antonio).

[B42] White R. B. Bierinx A. S. Gnocchi V. F. Zammit P. S. (2010). Dynamics of muscle fibre growth during postnatal mouse development. BMC Dev. Biol. 10, 21. 10.1186/1471-213X-10-21 20175910 PMC2836990

[B43] Xu J. Strasburg G. M. Reed K. M. Bello N. M. Velleman S. G. (2023). Differential effects of temperature and mTOR and Wnt-planar cell polarity pathways on syndecan-4 and CD44 expression in growth-selected Turkey satellite cell populations. PLoS One 18 (2), e0281350. 10.1371/journal.pone.0281350 36735684 PMC9897570

[B44] Xu J. Strasburg G. M. Reed K. M. Velleman S. G. (2021). Response of Turkey pectoralis major muscle satellite cells to hot and cold thermal stress: effect of growth selection on satellite cell proliferation and differentiation. Comp. Biochem. Physiol. A Mol. Integr. Physiol. 252, 110823. 10.1016/j.cbpa.2020.110823 33148517

[B45] Xu J. Strasburg G. M. Reed K. M. Velleman S. G. (2022). Temperature and growth selection effects on proliferation, differentiation, and adipogenic potential of Turkey myogenic satellite cells through Frizzled-7-mediated Wnt planar cell polarity pathway. Front. Physiol. 13, 892887. 10.3389/fphys.2022.892887 35677087 PMC9167958

[B46] Yablonka-Reuveni Z. Quinn L. S. Nameroff M. (1987). Isolation and clonal analysis of satellite cells from chicken pectoralis muscle. Dev. Biol. 119 (1), 252–259. 10.1016/0012-1606(87)90226-0 3025033 PMC4128172

[B47] Yamaguchi T. Suzuki T. Arai H. Tanabe S. Atomi Y. (2010). Continuous mild heat stress induces differentiation of mammalian myoblasts, shifting fiber type from fast to slow. Am. J. Physiol. Cell Physiol. 298 (1), C140–C148. 10.1152/ajpcell.00050.2009 19605738

